# Associations of Physical Activity, Sedentary Behaviour, Pain, Function and Quality of Life With Diabetes and Knee Osteoarthritis: Data From the Osteoarthritis Initiative

**DOI:** 10.1002/msc.70128

**Published:** 2025-06-10

**Authors:** Harvi F. Hart, Daniel K. White, Sonja M. Reichert, Joshua J. Stefanik

**Affiliations:** ^1^ Department of Kinesiology Michigan State University East Lansing Michigan USA; ^2^ School of Physical Therapy Western University London Canada; ^3^ Department of Physical Therapy University of Delaware Newark Delaware USA; ^4^ Department of Family Medicine Schulich School of Medicine and Dentistry Western University London Canada; ^5^ Primary Care Diabetes Support Program St Joseph's Health Care London London Canada; ^6^ Division of Rheumatology Boston University School of Medicine Boston University Boston Massachusetts USA; ^7^ Department of Physical Therapy, Movement, and Rehabilitation Sciences Northeastern University Boston Massachusetts USA

**Keywords:** comorbidity, diabetes mellitus, exercise, osteoarthritis, rehabilitation, sedentary behaviour

## Abstract

**Objective:**

To investigate the relation of radiographic knee osteoarthritis (RKOA) and type 2 diabetes mellitus (T2DM) to physical activity, pain, physical function, and quality of life.

**Methods:**

Data on physical activity (light and moderate‐vigorous physical activity minutes/day, steps/day, sedentary time percent of wear time), pain, physical function (Western Ontario and McMaster Index, walking speed during 20‐m walk) and quality of life (SF‐12) from the Osteoarthritis Initiative at the 48‐month visit were included. Participants (*n* = 1788) were categorised into no RKOA or T2DM, RKOA‐alone, T2DM‐alone, and RKOA and T2DM. Multivariable regression models, adjusted for age, sex, and BMI, assessed the relationship of disease status to outcomes.

**Results:**

Compared to RKOA‐alone, RKOA and T2DM were associated with lower moderate‐vigorous physical activity (Coefficient: 4 min/day, 95% CI: [−7, −1]) and steps/day (−817 steps/day [−1291, −343]) and higher sedentary time percent (1.3%/day [−0.2, 2.8]). No significant differences were found in light physical activity (−11 min/day [−25, 2]). The RKOA and T2DM groups reported greater pain (1.0 [0.4, 1.6]) and functional limitations (3 [1, 5]), slower walking speed (−0.09 m/s [−0.12, −0.05]), and worse quality of life (−3.2 [−4.8, −1.6]). T2DM‐alone was also associated with lower physical activity and worse quality of life than RKOA‐alone.

**Conclusions:**

The comorbidity of RKOA and T2DM and T2DM‐alone were associated with lower physical activity and worse quality of life than RKOA‐alone. Management should address both conditions concurrently rather than in isolation.

## 1 Introduction

Knee osteoarthritis (OA) and type 2 diabetes mellitus (T2DM) are prevalent chronic conditions that affect millions of individuals worldwide (Guariguata et al. 2014; Vos et al. 2012). Both conditions are associated with significant morbidity, reduced quality of life and mortality (Cleveland et al. 2019; Kawano et al. 2015; Losina et al. 2011; Solli et al. 2010). Knee OA and T2DM often coexist (Louati et al. 2015; Quiñones et al. 2019) and together lead to health challenges and exacerbate the burden beyond the presence of either disease alone (Calders and Van Ginckel 2018; King et al. 2022).

Daily activity, including physical activity and sedentary behaviour, is the cornerstone for managing chronic conditions, including knee OA and T2DM (Colberg et al. 2016; McAlindon et al. 2014). Engaging in regular physical activity is associated with improved pain, physical function and quality of life in individuals with knee OA (Kraus et al. 2019; Vitaloni et al. 2019), and enhanced disease outcomes, including better glycaemic control and reduced insulin resistance in individuals with T2DM (Sigal et al. 2018). On the other hand, sedentary behaviour is linked to musculoskeletal pain conditions, including knee pain and arthritis (Dzakpasu et al. 2021). Being less sedentary can improve physical function in those with knee OA (Lee et al. 2015) and lower cholesterol, insulin levels and waist circumference in individuals with T2DM (Koster et al. 2017). Unfortunately, individuals with knee OA or T2DM often report lower physical activity than those without these conditions (Cichosz et al. 2013; Kennerly and Kirk 2018; Lee et al. 2015).

Individuals with the comorbidity of knee OA and T2DM report lower self‐reported physical activity than those with knee OA alone (Seow et al. 2024). Another study reports that, after adjusting for covariates, T2DM in individuals with knee OA have a lower average daily step count and spend less time in light physical activity (Fujita et al. 2023), but but show no association with moderate to vigorous physical activity (Fujita et al. 2023). In addition to being less physically active, individuals with the comorbidity of knee OA and T2DM also appear to be more sedentary. Relative to controls without knee OA and T2DM, individuals with the comorbidity of knee OA and T2DM, as well as those with T2DM, report more sedentary behaviour (Cichosz et al. 2013; Kennerly and Kirk 2018; Lee et al. 2015; Wallis et al. 2013).

Comorbidities can exacerbate the impact of OA (Breedveld 2004), with the presence of at least one comorbidity associated with heightened levels of pain and lower self‐reported and performance‐based function in people with knee or hip OA (Calders and Van Ginckel 2018). In those with the comorbidity of knee OA and T2DM, some studies report increased knee pain intensity (Alenazi, Alshehri, et al. 2020; Alenazi, Obaidat, et al. 2020; Eitner et al. 2021; Eitner et al. 2017; Reeuwijk et al. 2010) and patient‐reported functional limitations (Reeuwijk et al. 2010). While some report no associations with pain or patient‐reported functional limitations but report associations with depressive symptoms and insomnia (Zullig et al. 2015). Another study reports the relationship between the comorbidity of knee OA and T2DM and worse performance‐based function; however, this is relative to those without knee OA or T2DM alone (Seow et al. 2024). Given the inconsistent findings from previous studies and the unique challenges that individuals with the comorbidity of T2DM and knee OA face in managing these two chronic conditions and the overall impact on their well‐being (King et al. 2022), further work is warranted to evaluate patient‐reported and performance‐based outcomes in individuals with the comorbidity of knee OA and T2DM relative to those with knee OA.

To understanding pain, physical function, and health‐related quality of life (HRQoL) in those with T2DM alone compared with those with knee OA alone may provide valuable insights. This comparison can help identify specific needs and challenges unique to each group, leading to more targeted and effective interventions. By distinguishing each condition separately and in combination, we can develop better management strategies to improve overall patient outcomes and quality of life. Therefore, this study investigated the relation of radiographic knee OA (RKOA) and T2DM disease status to daily activity (including physical activity and sedentary time), pain, physical function and HRQoL.

## 2 Methods

### 2.1 Study Population

This study used the data from the open‐access Osteoarthritis Initiative (OAI) study. The OAI is a prospective observational cohort study of 4796 adults aged 45–79 years, who have or are at risk of knee OA. Participants were recruited from four clinical sites in the US: Maryland, Pennsylvania, Rhode Island and Ohio. Full details of the study are available at https://nda.nih.gov/oai/. In a sub‐cohort of OAI participants, accelerometer data were available at the 48‐month study visit. In the present study, we included participants with four or more valid days of accelerometer monitoring who had knee radiographs and completed the Charlson Comorbidity Index at the 48‐month study visit. The data set includes bilateral posteroanterior fixed‐flexion knee radiographs from participants collected at the 48‐month study visit. In the present study, the Kellgren and Lawrence grades obtained from Project 15 were used. The Kellgren and Lawrence classification system was used to classify radiographic knee osteoarthritis (RKOA) severity. The presence of RKOA was defined as Kellgren and Lawrence grade ≥ 2 in either knee. A single question from the Charlson Comorbidity Index on whether the participant had diabetes (high blood sugar) was used to classify T2DM diagnoses. Participants were categorised into four groups based on their RKOA and T2DM disease status: (i) no RKOA or T2DM, (ii) RKOA‐alone, (iii) T2DM‐alone, and (iv) RKOA and T2DM. This study did not require institutional review board approval as it involved the use of publicly available open‐access data.

### 2.2 Daily Activity

Physical activity was monitored using ActiGraph GT1M uniaxial accelerometers (ActiGraph; Pensacola, FL) over 7 days. Physical activity variables were synthesised from minute‐by‐minute accelerometer counts, aggregated on a day‐by‐day basis and finally aggregated to the participant level. Activity count/minute was determined using the cut points proposed by Troiano and colleagues (Troiano et al. 2008). In the present study, we included the mean minutes per day spent in light and moderate to vigorous intensity physical activity levels and the average number of steps taken per day for each participant. Additionally, average daily sedentary time (calculated as mean daily activity monitor wear minutes minus total physical activity minutes) and the average percentage of daily time spent sedentary were included.

### 2.3 Pain

Knee pain was assessed using the Western Ontario and McMaster Universities Index (WOMAC) pain scale (Bellamy et al. 1988). The score ranges from 0 to 20, with higher scores indicating worse pain.

### 2.4 Physical Function

Patient‐reported functional limitation was assessed using the WOMAC function scale (Bellamy et al. 1988). The score ranges from 0 to 68, with higher scores indicating worse functional limitations. The performance‐based function was assessed with walking speed during the 20‐m walk test. Participants were instructed to walk at their usual speed over a marked 20‐m course in an unobstructed and dedicated corridor. Participants were allowed to walk (i.e., could take three more steps) after they crossed the 20‐m mark. A digital stopwatch was used to record the time taken to complete the test. The timing began at the initial movement from standing at the start and stopped when they crossed the 20‐m mark. Walking speed in m/s was calculated by dividing the total distance (20 m) by the total time to complete the walk test (seconds).

### 2.5 HRQoL

The 12‐item Short‐Form Health Survey evaluated physical and mental HRQoL (Ware et al. 1996). Physical component summary and mental component summary scale scores ranged from 0 to 100, with higher scores indicating better physical and emotional functioning, respectively.

### 2.6 Statistical Analysis

Participants were categorised into four groups based on their RKOA and T2DM disease status: (i) no RKOA or T2DM, (ii) RKOA‐alone, (iii) T2DM‐alone and (iv) RKOA and T2DM. Participant characteristics are reported as means and standard deviations, or percentages, as appropriate. Using multivariable linear regression models, we tested the relation of disease status groups to daily activity, pain, physical function, and HRQoL in separate models. The RKOA‐alone group served as the reference group in the models, as the primary analysis focus was a comparison of the RKOA and T2DM groups with the RKOA‐alone group. RKOA or T2DM and T2DM‐alone groups were also compared relative to the reference group. Analyses were adjusted for age, sex and body mass index. Unstandardised beta with 95% confidence intervals is presented. Data were analysed using STATA (version 17).

## 3 Results

### 3.1 Participants Characteristics

**FIGURE 1:**
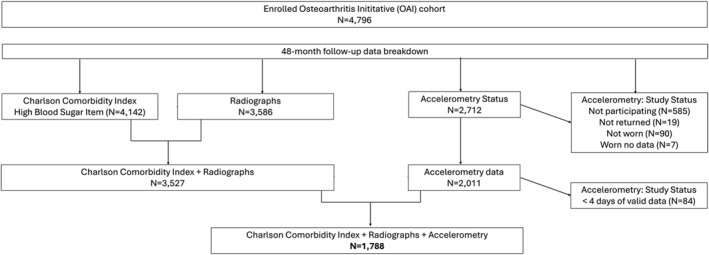
Flow chart of the OAI participants' selection.

In the present study, 1788 participants were included (Figure 1). Bilateral radiographs were available in 1721, and 67 only had unilateral radiographs. There were 638 participants in the no RKOA or T2DM group, 963 participants in the ROA‐alone group, 61 in the T2DM‐alone group, and 126 in the RKOA and T2DM groups (Table 1).

**TABLE 1:** Participants characteristics.

	RKOA‐alone (*n* = 963)	No RKOA or T2DM (*n* = 638)	T2DM‐alone (*n* = 61)	RKOA + T2DM (*n* = 126)
Demographics				
Age, years	66 ± 9	63 ± 9	65 ± 10	68 ± 8
Body mass index, kg/m^2^	29 ± 5	27 ± 5	30 ± 5	31 ± 5
Women, % (*n*)	55(532)	57 (363)	54 (33)	53 (67)
Daily activity				
Light physical activity, minutes/day	281 ± 77	286 ± 75	258 ± 83	261 ± 89
Moderate‐vigorous physical activity, minutes/day	17 ± 19	21 ± 21	11 ± 13	10 ± 13
Step count, steps/day	6019 ± 2822	6721 ± 3017	5223 ± 2778	4662 ± 2511
Proportion of time spent in sedentary behaviour, percent/day	66 ± 9	66 ± 8	69 ± 10	69 ± 10
Sedentary time, minutes/day	590 ± 19	591 ± 19	600 ± 94	591 ± 79
Pain and physical function				
WOMAC pain (0–20)	3 ± 4	2 ± 3	3 ± 4	4 ± 4
WOMAC physical function (0–68)	9 ± 11	5 ± 8	11 ± 14	13 ± 14
Walking speed, m/s	1.33 ± 0.20	1.37 ± 0.20	1.28 ± 0.24	1.21 ± 0.22
HRQoL				
Physical health (0–100)	48 ± 9	51 ± 8	45 ± 10	44 ± 10
Mental health (0–100)	54 ± 8	53 ± 9	53 ± 10	54 ± 9

*Note:* Data are presented as mean ± standard deviation unless otherwise stated.

Abbreviations: HRQoL, health‐related quality of life; ROA, radiographic knee osteoarthritis; T2DM, type 2 diabetes; WOMAC, Western Ontario and McMaster Arthritis Index.

### 3.2 Association of Disease Status To Outcomes

**FIGURE 2:**
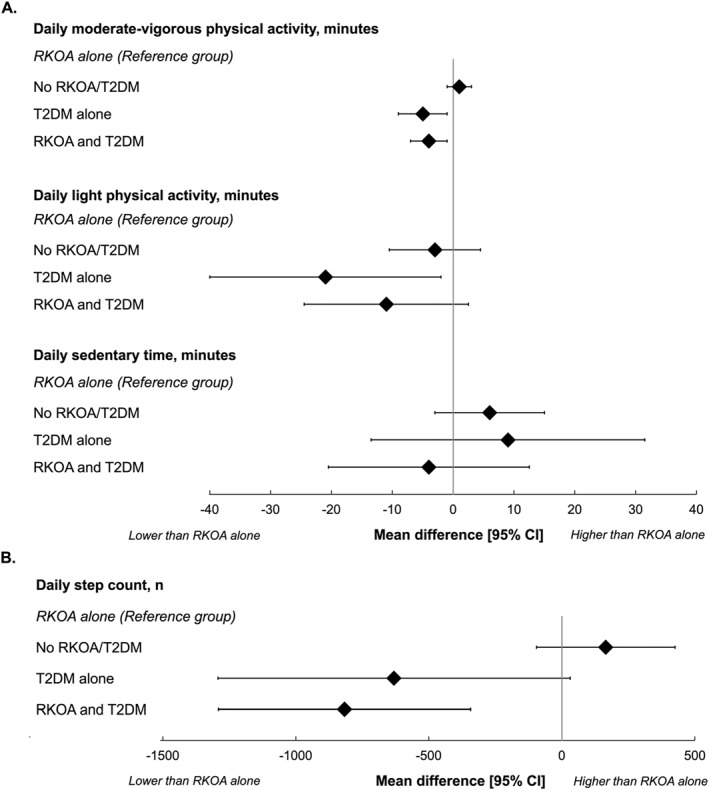
Daily minutes of moderate‐vigorous physical activity, light physical activity, and sedentary behaviour (A.), and step count (B.) in individuals without radiographic knee osteoarthritis (OA) or type 2 diabetes mellitus (T2DM), those with T2DM alone, and those with RKOA and T2DM relative to RKOA alone. Data are presented as coefficients with 95% confidence intervals (CI).

**FIGURE 3:**
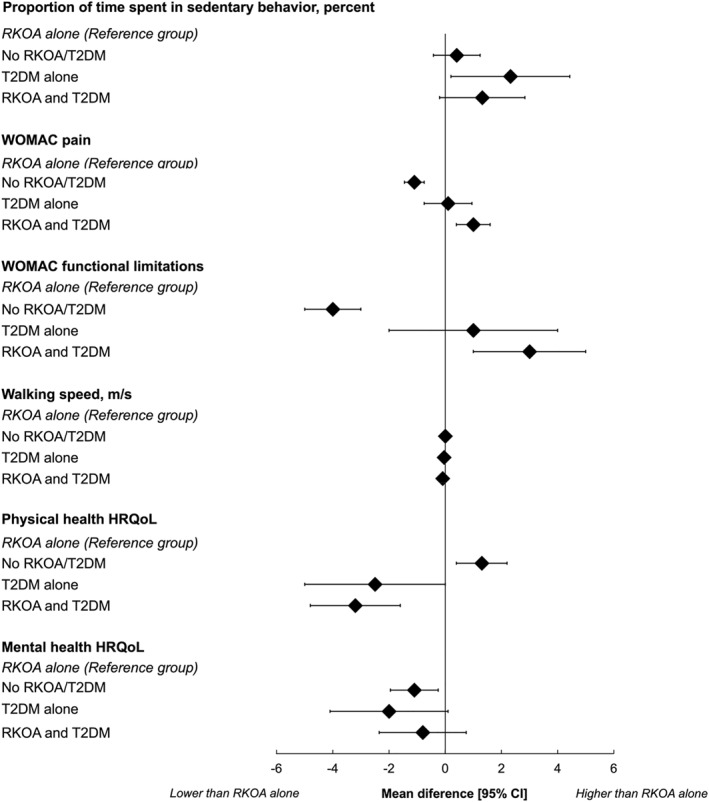
The daily proportion of time spent sedentary, pain, physical function (the Western Ontario and McMaster Universities Arthritis Index [WOMAC] functional limitations subscale and walking speed), and health‐related quality of life (HRQoL) in individuals without radiographic knee osteoarthritis (RKOA) or type 2 diabetes mellitus (T2DM), those with T2DM only, and those with both RKOA and T2DM compared to those with RKOA only. Data are presented as coefficients with 95% confidence intervals (CIs).

Individuals in the RKOA and T2DM group participated in less moderate‐to‐vigorous physical activity (B [95% CI]: −4 min/day [−7 to −1]), took fewer steps per day (−817 steps/day [−1291 to −343]), had a higher percentage of sedentary time (1.32 percent/day [−0.20 to 2.84]), had slower walking speed (−0.09 m/s [−0.12 to −0.05]), had more pain (0.97 [0.37 to 1.58]) and functional limitations (3 [1 to 5]), and reported a poorer physical HRQoL (−3.2 [−4.8 to −1.6]) when compared to the group without RKOA or T2DM (Table 1, Figures 2 and 3).

Individuals in the T2DM‐alone group engaged in less light physical activity (−21 min/day [−2 to 40]) and moderate‐to‐vigorous physical activity (−5 min/day [−9 to −1]), took fewer steps per day (−631 steps/day [−1293 to 31]) and reported lower physical HRQoL (−2.5 [−4.7 to −0.3]) relative to those without RKOA or T2DM (Table 1, Figures 2 and 3).

On the other hand, the group without RKOA or T2DM had lower levels of pain (−1.1 [−0.8 to −1.5]) and functional limitations (−4 [−5 to −3]), higher physical HRQoL (1.3 [0.4 to 2.2]), but poorer mental HRQoL (−1.1 [−1.9 to −0.2]) compared to the group with ROA‐alone (Table 1, Figures 2 and 3).

## 4 Discussion

Our study highlights that individuals with the comorbidity of RKOA and T2DM exhibit less physical activity and spend more time in sedentary behaviour compared with those with RKOA alone. They also reported greater pain, poorer physical function and lower physical HRQoL. Individuals with T2DM alone also show less physical activity, more sedentary behaviour and worse physical HRQoL than those with RKOA alone.

The presence of comorbidities has been associated with reduced engagement in light and moderate‐to‐vigorous physical activity (Barker et al. 2019; Brawner et al. 2016). A recent study found that individuals with knee OA and T2DM have lower self‐reported physical activity (Seow et al. 2024). Our findings indicate that individuals with both RKOA and T2DM engage in fewer minutes/day in moderate‐to‐vigorous physical activity and take fewer steps/day compared with those with RKOA alone. There were no differences observed in light physical activity minutes. This is somewhat consistent with a previous study reporting fewer average daily steps in those with T2DM and knee OA. Additionally, the study found that T2DM was associated with fewer light physical activity minutes, but not moderate‐to‐vigorous physical activity minutes (Fujita et al. 2023). Participants in the aforementioned study accumulated lower physical activity minutes and step count in both groups, RKOA with and without T2DM (Fujita et al. 2023), compared to the RKOA alone, and the RKOA and T2DM groups in the present study.

The World Health Organization recommends that adults accumulate at least 150 min of moderate‐to‐vigorous intensity physical activity per week (Bull et al. 2020), which translates to about 21 min per day. The physical activity guidelines for managing T2DM also align with the World Health Organization recommendations (Sigal et al. 2018). Except for the group with neither RKOA nor T2DM, none of the other groups met this recommendation. The lowest levels of moderate‐to‐vigorous physical activity were reported in those with both RKOA and T2DM (mean 10 min/day) and those with T2DM alone (mean 11 min/day). Meta‐analyses have reported an average of 131 min per week of moderate‐to‐vigorous physical activity in individuals with knee OA (Wallis et al. 2013). Our findings indicate that individuals with RKOA alone achieve 119 min per week, whereas those with both RKOA and T2DM achieve only 70 min per week. The average daily light physical activity minutes were similar between individuals with RKOA alone and those with both RKOA and T2DM. Those with T2DM alone accumulated the lowest average light physical activity minutes (261 min/day).

Individuals with RKOA and T2DM and those with knee OA alone took fewer steps per day than those with T2DM alone. Accumulating less than 6000 steps per day has been associated with increased functional limitations in individuals with or at risk of knee OA (White et al. 2014). Notably, neither the RKOA and T2DM group nor the T2DM alone group in the present study achieved the 6000 steps per day threshold.

Previous studies report that individuals with knee OA and T2DM typically spend about two‐thirds of their waking days being sedentary (Cichosz et al. 2013; Lee et al. 2015; Loprinzi 2014; van der Berg et al. 2016). In the present study, individuals across all groups spent approximately 9–10 h per day, or over two‐thirds of the day, being sedentary. The proportion of the day spent being sedentary was higher in individuals with RKOA and T2DM, and those with T2DM alone, compared to those with RKOA alone. Being physically active and less sedentary has been associated with health benefits (Koster et al. 2017; Kraus et al. 2019; Lee et al. 2015; Sigal et al. 2018). Our findings indicate that individuals with a combination of knee OA and T2DM, and those with T2DM alone, are less physically active and more sedentary throughout the day. Because of the cross‐sectional nature of the analysis, the causal relationship between physical activity, sedentary behaviour and disease status cannot be determined. It may be that a lack of physical activity contributes to the development of T2DM, and that the presence of both radiographic knee OA and T2DM further exacerbates inactivity.

Walking speed is an important indicator of functional status and overall health (Middleton et al. 2015). Osteoarthritis‐related walking difficulty has been reported as a significant predictor of T2DM‐related complications (Hawker et al. 2017). As anticipated, individuals with both RKOA and T2DM exhibited the slowest walking speed, whereas those without RKOA or T2DM exhibited the highest walking speed. Individuals with both RKOA and T2DM walked more slowly than those with RKOA alone. Notably, the average walking speed in individuals with both RKOA and T2DM was 1.21 m/s. A walking speed slower than 1.22 m/s is predictive of reduced physical activity capacity in individuals with or at risk of knee OA (Master et al. 2018).

Individuals with both RKOA and T2DM reported more pain and functional limitations than those with RKOA alone. The finding contrasts with a study that compared individuals with RKOA (Fujita et al. 2023), but is consistent with other previous research reporting higher pain severity in patients with both knee OA and T2DM (Alenazi, Alshehri et al. 2020; Eitner et al. 2017). Interleukin‐6, a systemic inflammatory marker, has been implicated in the aetiology of T2DM and RKOA (Bowker et al. 2020; Livshits et al. 2009) and is associated with worsening knee pain (Stannus et al. 2013). Among patients with end‐stage knee OA, higher levels of interleukin‐6 have been reported in those with T2DM than in those without (Eitner et al. 2017). It is plausible that elevated levels of systemic inflammation in individuals with both T2DM and RKOA contribute to increased pain intensity, and this heightened pain may be contributing to inactivity, functional limitations and poorer HRQoL. However, this is speculative as this study only provides a snapshot of symptoms and physical function based on RKOA and T2DM disease status.

The present study has several limitations that ought to be acknowledged. First and foremost, the associations presented are derived from cross‐sectional data; thus, causation cannot be inferred. In the present study, participants were categorised based on radiographic knee OA and T2DM disease status. However, the OAI is a database of individuals with or at risk of OA, meaning that participants categorised as having no RKOA may not be true representatives of a no‐knee OA group. Additionally, 67 individuals had radiographs available for only one knee, and of these, 15 were classified in the No RKOA or T2DM group. Some of these individuals may have had RKOA in the other knee, which could have led to misclassification and potentially influenced the findings.

Additionally, physical activity and sedentary behaviour may differ in individuals with well‐managed and uncontrolled T2DM; however, this was not considered in the present study. The T2DM‐alone and T2DM and RKOA groups were smaller than the other study groups. Thus, we could not analyse the data in women and men separately. Sex‐based analysis in future studies could provide valuable insights and inform tailored treatment approaches for health outcomes associated with T2DM and RKOA.

## 5 Conclusion

Individuals with the comorbidity of RKOA and T2DM, as well as those with T2DM without RKOA, exhibit lower levels of daily physical activity and spent a higher proportion of their day being sedentary compared with those with RKOA alone.

## Author Contributions

All authors made significant contributions to each of the following: (i) substantial contribution to conception and design, execution or analysis and interpretation of data, (ii) drafting the article or revising it critically, and (iii) reading and approval of the final version.

## Ethics Statement

The authors have nothing to report.

## Consent

The authors have nothing to report.

## Conflicts of Interest

The authors declare no conflicts of interest.

## Supporting information

Supplementary Material

## Data Availability

The data that support the findings of this study are openly available in The Osteoarthritis Initiative at https://nda.nih.gov/oai/.
