# Supplementary material for: Associations of Physical Activity, Sedentary Behaviour, Pain, Function and Quality of Life With Diabetes and Knee Osteoarthritis: Data From the Osteoarthritis Initiative
Source: Musculoskeletal Care. 2025 Jun 10;23(2):e70128. doi: 10.1002/msc.70128 (PMC12152236; doi:10.1002/msc.70128)
Supplement: Supplementary file 1 — Supplementary Material [file MSC-23-e70128-s001.docx]

| **Supplementary Material 1**. Distribution of radiographic knee osteoarthritis (RKOA) status by type 2 diabetes mellitus (T2DM) disease status | | | |
| --- | --- | --- | --- |
|  | No RKOA | Unilateral RKOA | Bilateral RKOA |
| No T2DM | 638^^^ | 475^‡^ | 488 |
| T2DM | 61^†^ | 52^§^ | 74 |
| Total | 699 | 527 | 564 |
| ^^^n=14 based on unilateral radiograph, ^†^n=1 based on unilateral radiograph, ^‡^n=47 based on unilateral radiograph, ^§^n=5 based on unilateral radiograph | | | |
